# The use of gelatine in wound ballistics research

**DOI:** 10.1007/s00414-018-1831-7

**Published:** 2018-04-25

**Authors:** D. J. Carr, T. Stevenson, P. F. Mahoney

**Affiliations:** 1 Impact and Armour Group, Centre for Defence Engineering, Cranfield University, Defence Academy of the UK, Swindon, SN6 8LA UK; 2 Defence and Security Accelerator, Porton Down, Salisbury, SP4 0JQ UK; 3 Cranfield Forensic Institute, Cranfield University, Defence Academy of the UK, Swindon, SN6 8LA UK

**Keywords:** Gelatine, Bullets, Temporary and permanent cavities, Lethality and survivability

## Abstract

Blocks of gelatine are used in both lethality and survivability studies for broadly the same reason, i.e. comparison of ammunition effects using a material that it is assumed represents (some part of) the human body. The gelatine is used to visualise the temporary and permanent wound profiles; elements of which are recognised as providing a reasonable approximation to wounding in humans. One set of researchers aim to improve the lethality of the projectile, and the other to understand the effects of the projectile on the body to improve survivability. Research areas that use gelatine blocks are diverse and include ammunition designers, the medical and forensics communities and designers of ballistic protective equipment (including body armour). This paper aims to provide an overarching review of the use of gelatine for wound ballistics studies; it is not intended to provide an extensive review of wound ballistics as that already exists, e.g. Legal Med 23:21–29, 2016. Key messages are that test variables, projectile type (bullet, fragmentation), impact site on the body and intermediate layers (e.g. clothing, personal protective equipment (PPE)) can affect the resulting wound profiles.

## Why are gelatine blocks used for wound ballistics studies?

Use of gelatine blocks can result in similar projectile depth of penetration (DoP) and permanent damage to that observed in soft tissue (living and cadaveric) [1–4]. In modern wound ballistics studies, either 10 or 20% gelatine (by mass) blocks conditioned to 4 and 10 °C respectively are used. Gelatine blocks of 10% concentration by mass (conditioned to 4 °C) resulted in DoP to within 3% for selected bullets compared to those in living swine thigh tissue [4, 5] and similar DoP to swine torso [6]. A recent comparison of wounds caused by 4.8 mm diameter ball bearings (1150 ± 5 m/s) in 10% (by mass) gelatine and the legs of anesthetised swine reported similar trajectories in both targets, DoP within 1% and the pattern of temporary cavity formation and collapse being similar, but the maximum size and duration larger (12%) and longer (24%) in gelatine [7]. Gelatine blocks of 20% (by mass, usually conditioned to 10 °C) are used by some in the wound ballistic testing community and are often referred to as “NATO gelatine” [8–11]. However, there is no NATO standard for gelatine and therefore “20% gelatine (by mass)” should be used. That 20% gelatine is stiffer than 10% gelatine and that DoPs are shorter in 20% gelatine is clear, although few people compare the two [6, 12].

Irrespective of concentration, type A gelatine^1^ with 250 to 300 Bloom is usually used [13, 14]. The resulting block is translucent allowing the interaction between the projectile and block to be imaged using high-speed video to view the formation and collapse of the temporary cavity. X-rays, CT scans and the use of dyes can aid the viewing of the permanent cavity [13, 15, 16]. Physical dissection, measurement and still photography can aid in the measurement of the depth of the penetration of the projectiles or fragmented projectiles and the analysis of the permanent cavity, which is typically in the form of the wound tract and fissures [6, 15, 17–20].

Alternative synthetic materials have been used and reportedly produce similar results as gelatine without the need to condition at a particular temperature (e.g. PermaGel^™^, Clear Ballistics Gel^®^). Claimed advantages include the ability to melt and re-use these materials without detrimental effect to the physical and mechanical properties (within limits). However, literature has reported that these materials produce different DoP and damage when compared to gelatine blocks [21, 22]. Evidence of ageing after one re-melt has also been reported, and burning within the blocks (which is not observed in gelatine blocks) is observed post-testing due to the composition of the material [21, 23].

Several researchers have commented on the method to manufacture gelatine blocks and the variables that affect consistency within and among blocks. Issues discussed include water temperature [13, 24], water acidity [24, 25], batch [21, 24] and longevity [13, 24–26]. Consistency of individual gelatine blocks can be assessed after temperature conditioning. This typically involves shooting a steel ball bearing (BB) into the block and measuring the DoP. Some researchers use 4.5 mm diameter BBs at relatively slow velocities [24–27] and others use 5.5 mm diameter BBs at faster velocities [21, 23, 28]. Based on DoP data of such non-deforming and non-yawing projectiles, one might expect that minimal variation would be seen in DoP measurements for deforming and/or fragmenting and/or yawing projectiles and other measures of damage in the gelatine block, but this is not the case.

Although researchers have considered the variability of gelatine manufacture, few consider the variability of the projectiles used. That bullets’ physical, mechanical and ballistic properties can vary is widely recognised within the ballistics community, but is rarely discussed in the literature [29, 30]. Use of a single batch (quarantined if necessary) and identification of composition (using SEM-EDS) and microhardness are recommended [21, 23, 28].

## Incorporating bone and bone simulants

Many researchers have recognised that not all ballistic impacts on the human body interact only with soft tissue such as muscle. Human and animal bones are often combined with gelatine to produce a target with improved biofidelity. Examples include the use of femurs (e.g. human, swine, deer) embedded in gelatine to represent human lower extremities, e.g. [31–34], combinations of thoracic sections with gelatine, e.g. [27, 35], use of human skulls, e.g. [36, 37] or use of flat bones to represent the skull, e.g. [19]. A number of polymeric bone simulants exist and have been used to represent skulls, e.g. [19, 38–40], and other bones [41–43]; these may be anatomically accurate or a simple geometric representation.

## Projectiles

Two types of projectiles are usually considered (i) bullets and (ii) fragmentation (typically ball bearings).

### Bullets

**Fig. 1:**
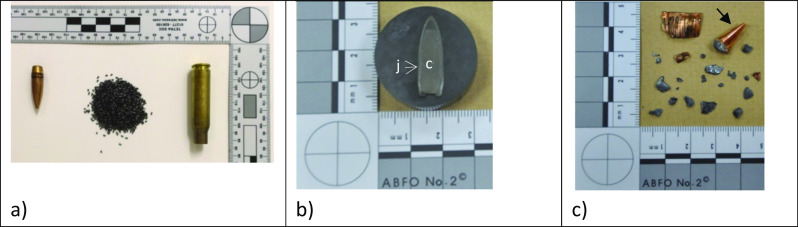
Ammunition. **a** Ammunition components: bullet, propellant and cartridge case which contains the primer in the base (left to right). **b** 7.62 x 39 mm bullet (fired from an AK47 rifle) cut in half lengthwise, mounted in Bakelite and polished; the core (c) and jacket (j) can be seen. **c** 5.56 mm NATO bullet (fired from a military rifle) fragments recovered from a block of gelatine; note the tip of bullet (arrowed)

Understanding how different types of bullets interact with a target is critical to understanding wound ballistics, and therefore understanding bullet construction is important [44]. A round of ammunition comprises of four basic component parts: (i) the cartridge case, (ii) the primer, (iii) the propellant and (iv) the bullet which is the part that leaves the gun and enters the target (Fig. 1a). Bullets may be fired from a handgun (pistol) or a rifle. Pistol bullets are usually slower than rifle bullets (e.g. 9 mm Luger bullet from a GLOCK pistol 350 m/s, 7.62 mm bullet from an AK47 rifle 730 m/s). Bullets that contain central cores covered by a layer of material are jacketed bullets. The jackets of full metal jacket (FMJ) bullets typically cover the bullet from the tip down to the base, leaving part of the core at the base exposed (Fig. 1a, b). FMJ bullets tend not to deform greatly during impact through soft tissues, often resulting in the complete perforation of targets; however, some FMJ bullets deform and/or fragment (Fig. 1c). If FMJ bullets interact with a relatively harder material (such as body armour) before entering soft tissues they may deform, the jacket may be stripped off and they may fragment. Partially jacketed bullets are typically jacketed from the base up, with part of the core left exposed (i.e. unjacketed) at the tip. This design encourages the expansion (deformed into a “mushroom” shape by the impact force) of bullets on impact, resulting in kinetic energy being dissipated sooner in a soft tissue penetration event as well as penetration depths shorter than those typical for FMJ bullets.

### Fragmentation

Fragmentation originates from a device such as a grenade or an improvised explosive device (IED). Typically, a metallic container is filled with explosive or a container is filled with metallic objects (such as nails, nuts and/or ball bearings) and explosive. On initiation, the explosive accelerates the fragmentation towards a target which typically suffers multiple impacts of varying DoPs. The injuries may combine the effects of blast and impacts from fragmentation [45].

## Wound ballistics

**Fig. 2:**
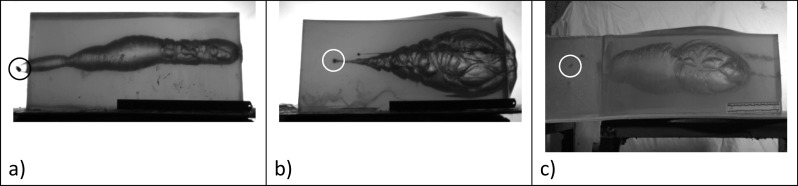
Typical stills from high-speed video footage illustrating extent of temporary cavitation in a 250 mm × 250 mm × 500 mm 10% (by mass) gelatine blocks conditioned to 4 °C (bullets circled). **a** 9 mm Luger FMJ [21]—a bullet fired from a pistol that tumbles through gelatine. **b** 0.223 Remington [6]—a non-military bullet fired from a rifle. This bullet expands, does not tumble and remains in the block. **c** 7.62 × 39 mm [23]—a military bullet fired from a rifle that tumbles; the bullet captured in a second block

How a projectile interacts with, penetrates into and perforates through (if it does) the human body depends on many factors including (i) the type of projectile and (ii) where on the body the projectile impacts. Wounding patterns are impossible to predict without knowing the full details of the incident, and even then, variability will exist. It is not the intention of this paper to provide an in-depth discussion of the physics of wound ballistics; that topic is covered in Kneubuehl’s classic text on the subject [44]; however, a short summary is required. Wounding occurs because kinetic energy is dissipated in the body due to the projectile interacting with it. When a bullet leaves a gun, it is spinning; on impacting the target, it de-accelerates. Depending on the bullet design and materials used, it may yaw in the body (tumble end over end), or it may expand; the bullet may also break-up (Fig. 1c). A temporary cavity is formed which collapses to leave permanent damage; the size of the temporary cavity is influenced by the elastic properties of the soft tissue type in which it forms and whether the bullet expands, fragments or tumbles (Fig. 2). If the bullet remains in the body then all of the kinetic energy of the impact event is transferred to the tissues (Fig. 2b).

**Fig. 3:**
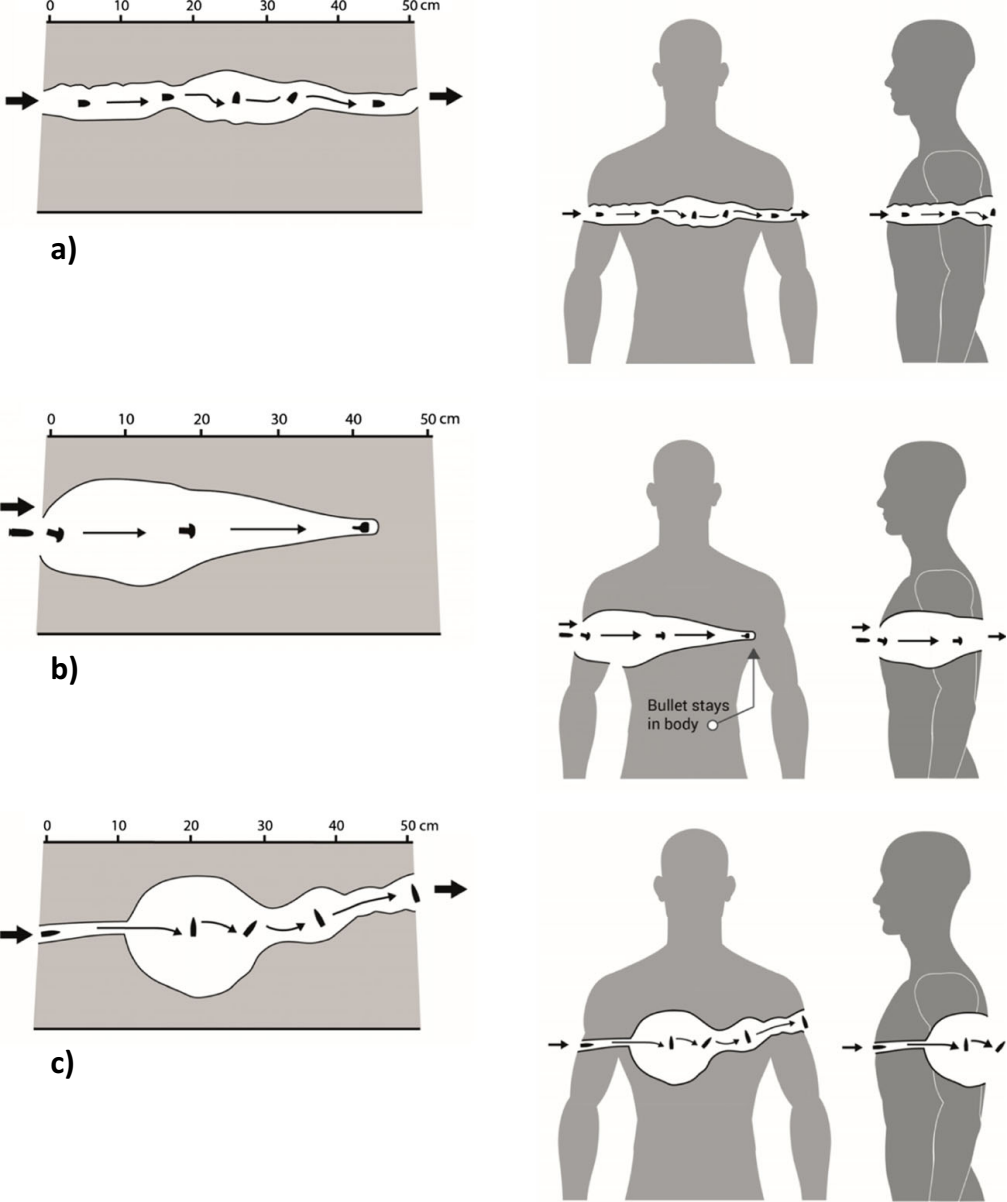
Typical temporary cavities in 10% (by mass) gelatine blocks and mapped onto the human body. **a** 9 mm FMJ with reference to [21]. **b** 0.233 Remington with reference to [6]. **c** 7.62 × 39 mm mild steel core (MSC) with reference to [19]

It is generally assumed there will be a small round entry hole and large irregular exit hole; numerous examples exist in medical and forensic case study journal articles and within experimental studies. A small round entry hole may be seen, but if the bullet hits the body side-on rather than front-on the entry hole will look very different. The appearance will also vary with the area of the body impacted. Figure 3, a modified version of which is used in the 2018 NHS Trauma Guidelines, demonstrates the differences that might be seen when the same bullet impacts the body on the lateral or anterior aspects.

**Fig. 4:**
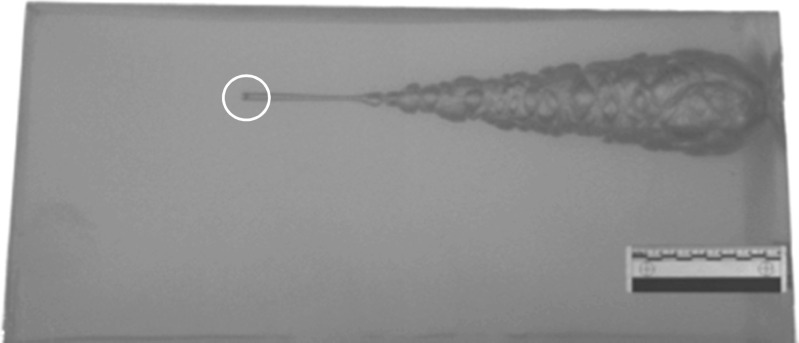
Example of a 5.5 mm diameter steel ball bearing (impact velocity = 654 m/s) penetrating a 250 mm × 250 mm × 500 mm gelatine block (10% by mass, conditioned to 4 °C). Maximum temporary cavity is shown and the final position of the ball bearing is circled.

That fragments (e.g. ball bearings which do not typically deform nor yaw) form a temporary cavity (albeit quite small in volume) as well as a permanent tract surprises some researchers not familiar with the field (Fig. 4).

## What to measure in wound ballistics studies?

Bullet muzzle velocity is often quoted, but it is arguable that the impact velocity at the target is more useful especially when considering the development or overmatching of PPE. Impact velocity can be equated to an estimated engagement distance.

With respect to damage in the gelatine blocks, researchers have used the DoP of the bullet as a key indicator of injury [6, 46]. However, the bullet is not always retained in a single block [6, 19, 20]. That the damage in the block should be considered within the context of the body segment of interest seems reasonable, but few actually discuss this [19, 21, 44]. Also of interest is the extent of the temporary cavity, the distance at which a yawing bullet yaws to maximum, a measure of fissure damage and any fragmentation of the bullet [5, 6, 15, 18–20, 47, 48]. Some researchers consider the amount of tissue removed during a surgical procedure; Jussila provided an interesting analysis of prior published data [49]. Wound profiles of temporary cavities allow different ammunition types to be compared, but it is always important to remember those profiles are in blocks of gelatine not in the human body; however, wound profiles from blocks can be mapped onto the body (Fig. 3) [15]. Rarely does anyone comment on the variability of such wound profiles [12, 21, 23, 28].

## Effect of intermediate layers such as clothing and personal ballistic protection

The effect of intermediate layers such as clothing has been considered in the context of increasing the possibility of infection in a wound [50–52], but also in the context of how such intermediate layers might affect wounding [19, 53, 54]. Thick, stiff clothing layers can affect the deformation of the bullet. This in turn can affect the formation of the temporary cavity and may result in more extensive bone fractures [53], larger wound sizes [55] and an increased quantity of fragmented ribs being pushed into the lungs [54]. Thin clothing layers do not appear to cause such issues [20].

Wounding behind PPE such as body armour and helmets is rarely discussed in the literature; this research area is at the interface of lethality and survivability studies. Published data suggests that some aspects of the wound profile can be affected by such PPE [19, 21, 35].

## Conclusions

Researchers that use gelatine blocks for lethality and survivability studies should minimise variability through sound experimental design and planning. The academic published literature in this area is in diverse journals, and practitioners within one discipline (e.g. medical science) should seek literature outside of their area (e.g. forensic science). Gelatine blocks should be validated for use, and that gelatine has strain rate sensitive properties should be considered. However, users should still expect variability in the measures used in wound profiles; thus, a suitable number of replicates are recommended. Different bullets result in different wound profiles due to how the bullet interacts with the target. Ammunition should be batch quarantined for experiments and the variability of the composition, microhardness and impact velocity considered. If the bullets within or between batches are of variable quality with respect to the materials and manufacturing standards used, this will only add to the variability reported in wound ballistics studies. The researcher must therefore consider bullet design and quality assurance. Intermediate layers may change the wound profile observed, and researchers in both the lethality and survivability areas should consider this.

## Footnotes

1 Type A gelatine is produced from acid-treated collagen. The gel (or jelly) strength of gelatine is traditionally referred to by *Bloom number*. To test the Bloom number, a 112 g sample of 6.67% *w*/*w* gelatine is prepared following a standardised time and temperature system. The sample is then brought to 10 °C before a plunger (12.5 mm in diameter) is pushed 4 mm into the gelatine. This is done by dropping shot into a cup until the plunger reaches the 4 mm depth required. The mass (in grams) of the shot required to achieve the 4 mm depth is the Bloom number.
